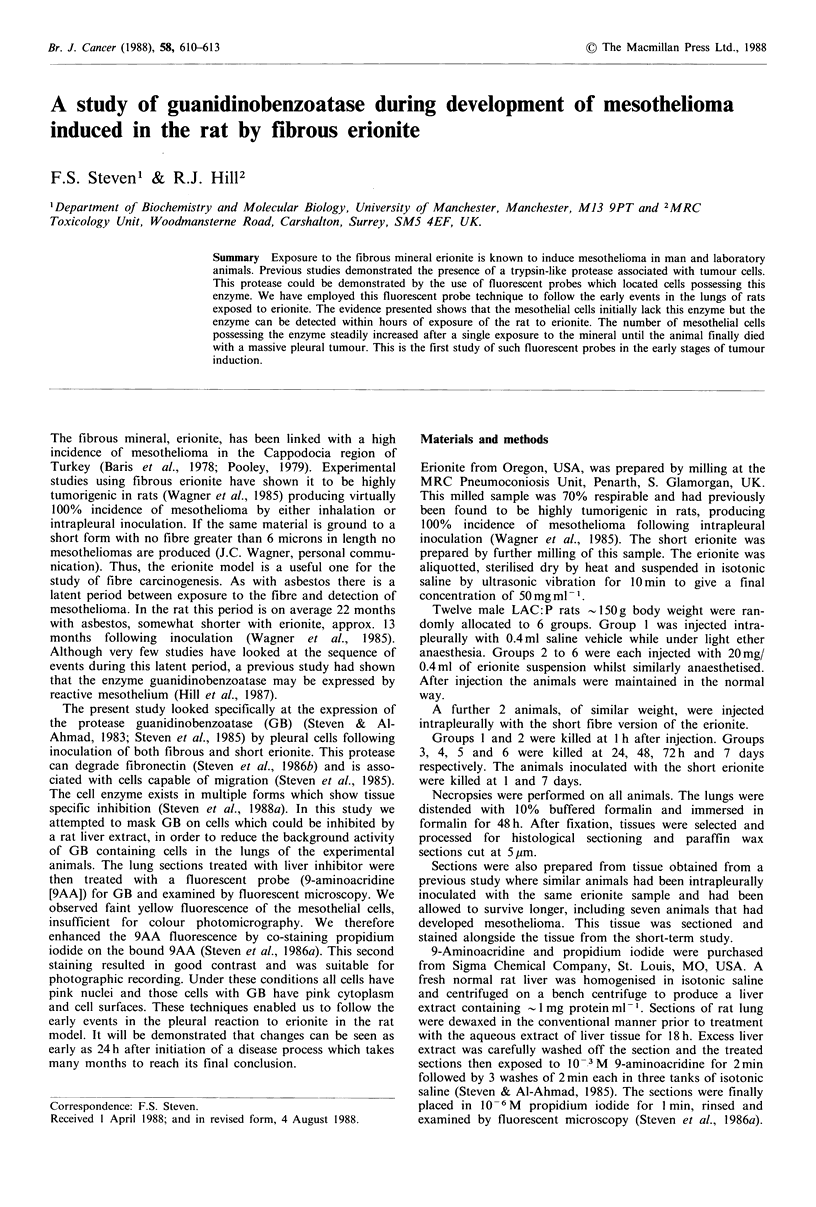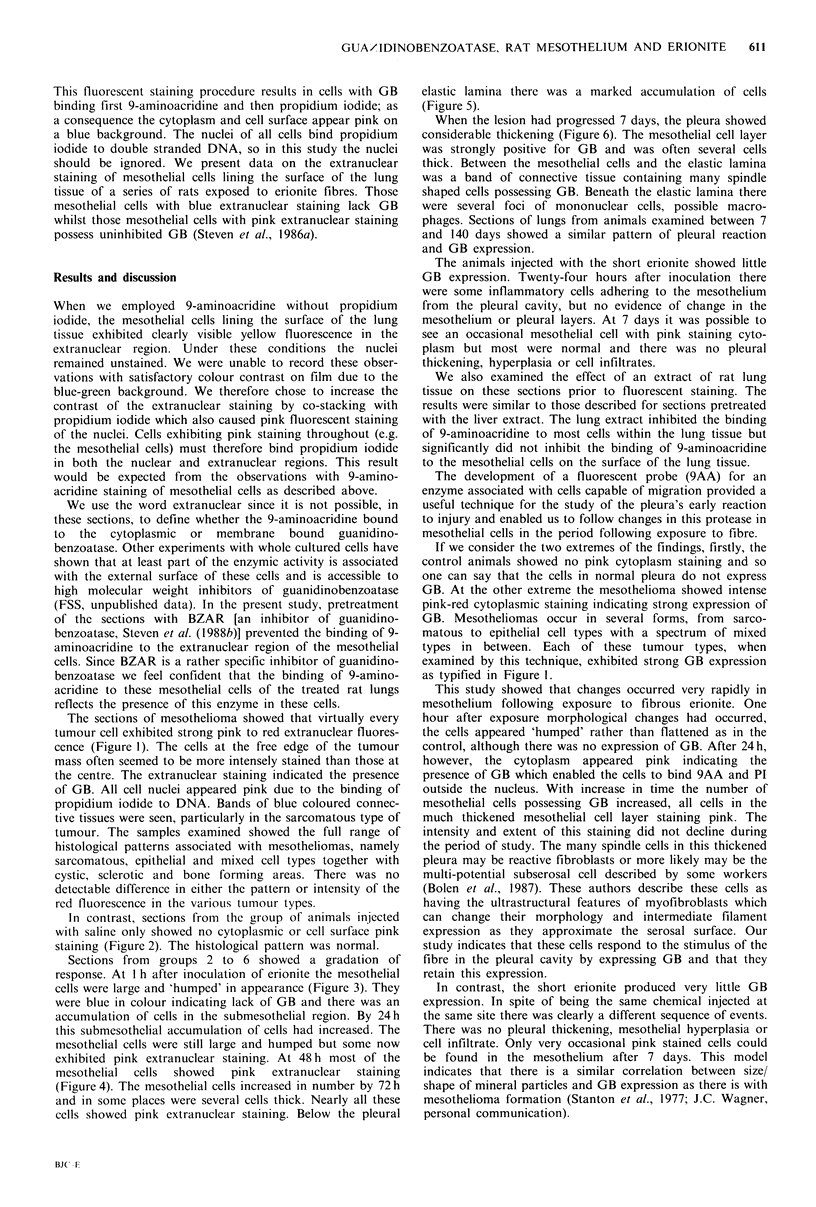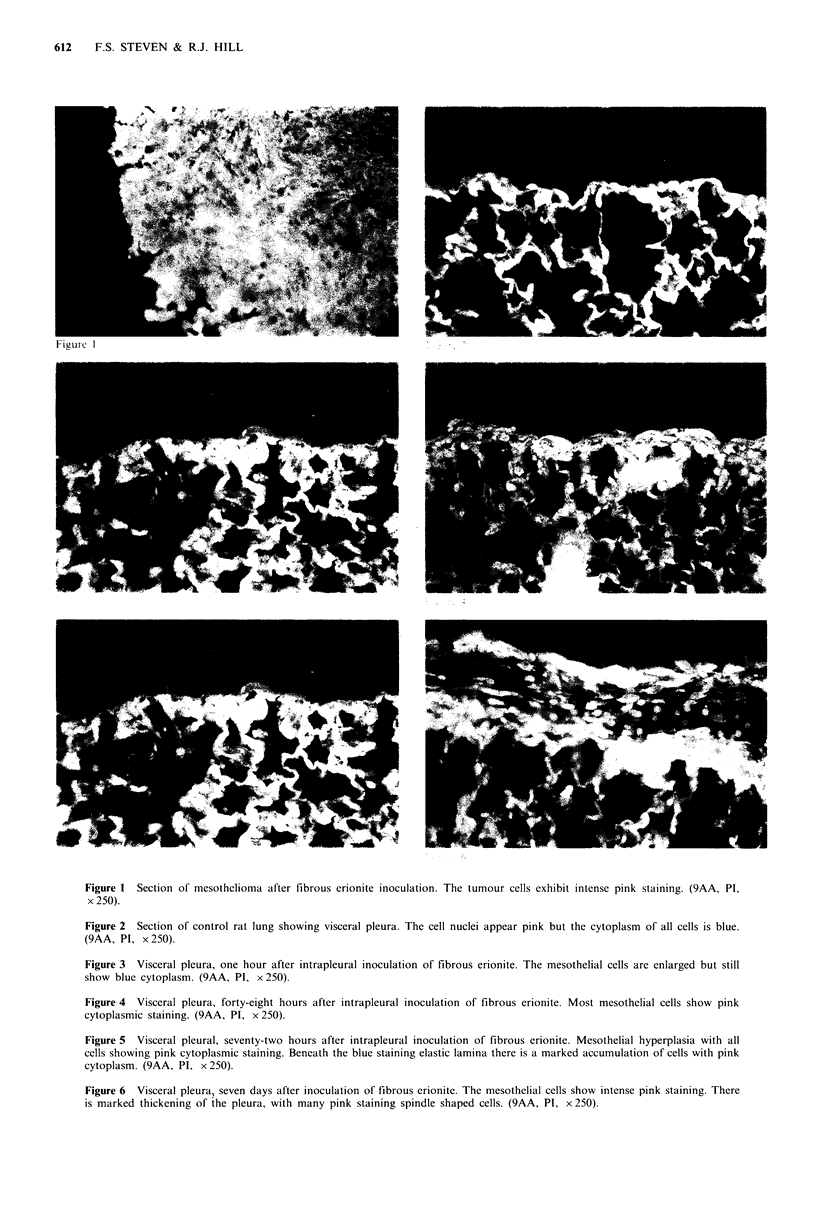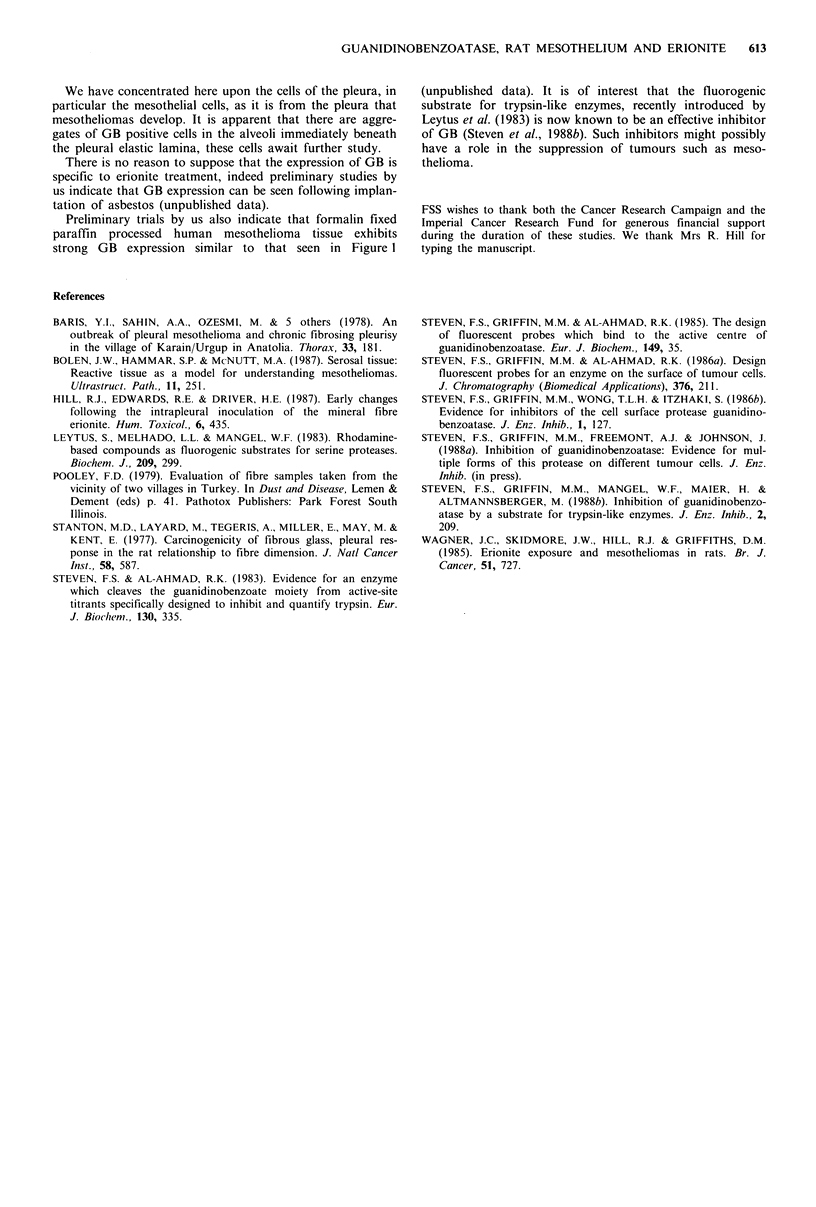# A study of guanidinobenzoatase during development of mesothelioma induced in the rat by fibrous erionite.

**DOI:** 10.1038/bjc.1988.269

**Published:** 1988-11

**Authors:** F. S. Steven, R. J. Hill

**Affiliations:** Department of Biochemistry and Molecular Biology, University of Manchester, UK.

## Abstract

**Images:**


*Br. J. Cancer* (1988), **58**, 610–613 © The Macmillan Press Ltd., 1988

### A study of guanidinobenzoatase during development of mesothelioma induced in the rat by fibrous erionite

F.S. Steven[1] & R.J. Hill[2]

[1] *Department of Biochemistry and Molecular Biology, University of Manchester, Manchester, M13 9PT and* [2] *MRC Toxicology Unit, Woodmansterne Road, Carshalton, Surrey, SM5 4EF, UK.*

**Summary** Exposure to the fibrous mineral erionite is known to induce mesothelioma in man and laboratory animals. Previous studies demonstrated the presence of a trypsin-like protease associated with tumour cells. This protease could be demonstrated by the use of fluorescent probes which located cells possessing this enzyme. We have employed this fluorescent probe technique to follow the early events in the lungs of rats exposed to erionite. The evidence presented shows that the mesothelial cells initially lack this enzyme but the enzyme can be detected within hours of exposure of the rat to erionite. The number of mesothelial cells possessing the enzyme steadily increased after a single exposure to the mineral until the animal finally died with a massive pleural tumour. This is the first study of such fluorescent probes in the early stages of tumour induction.

The fibrous mineral, erionite, has been linked with a high incidence of mesothelioma in the Cappodocia region of Turkey (Baris *et al.*, 1978; Pooley, 1979). Experimental studies using fibrous erionite have shown it to be highly tumorigenic in rats (Wagner *et al.*, 1985) producing virtually 100% incidence of mesothelioma by either inhalation or intrapleural inoculation. If the same material is ground to a short form with no fibre greater than 6 microns in length no mesotheliomas are produced (J.C. Wagner, personal communication). Thus, the erionite model is a useful one for the study of fibre carcinogenesis. As with asbestos there is a latent period between exposure to the fibre and detection of mesothelioma. In the rat this period is on average 22 months with asbestos, somewhat shorter with erionite, approx. 13 months following inoculation (Wagner *et al.*, 1985). Although very few studies have looked at the sequence of events during this latent period, a previous study had shown that the enzyme guanidinobenzoatase may be expressed by reactive mesothelium (Hill *et al.*, 1987).

The present study looked specifically at the expression of the protease guanidinobenzoatase (GB) (Steven & Al-Ahmad, 1983; Steven *et al.*, 1985) by pleural cells following inoculation of both fibrous and short erionite. This protease can degrade fibronectin (Steven *et al.*, 1986*b*) and is associated with cells capable of migration (Steven *et al.*, 1985). The cell enzyme exists in multiple forms which show tissue specific inhibition (Steven *et al.*, 1988*a*). In this study we attempted to mask GB on cells which could be inhibited by a rat liver extract, in order to reduce the background activity of GB containing cells in the lungs of the experimental animals. The lung sections treated with liver inhibitor were then treated with a fluorescent probe (9-aminoacridine [9AA]) for GB and examined by fluorescent microscopy. We observed faint yellow fluorescence of the mesothelial cells, insufficient for colour photomicrography. We therefore enhanced the 9AA fluorescence by co-staining propidium iodide on the bound 9AA (Steven *et al.*, 1986*a*). This second staining resulted in good contrast and was suitable for photographic recording. Under these conditions all cells have pink nuclei and those cells with GB have pink cytoplasm and cell surfaces. These techniques enabled us to follow the early events in the pleural reaction to erionite in the rat model. It will be demonstrated that changes can be seen as early as 24 h after initiation of a disease process which takes many months to reach its final conclusion.

Correspondence: F.S. Steven.
Received 1 April 1988; and in revised form, 4 August 1988.

### Materials and methods

Erionite from Oregon, USA, was prepared by milling at the MRC Pneumoconiosis Unit, Penarth, S. Glamorgan, UK. This milled sample was 70% respirable and had previously been found to be highly tumorigenic in rats, producing 100% incidence of mesothelioma following intrapleural inoculation (Wagner *et al.*, 1985). The short erionite was prepared by further milling of this sample. The erionite was aliquotted, sterilised dry by heat and suspended in isotonic saline by ultrasonic vibration for 10 min to give a final concentration of 50 mg ml$^{-1}$.

Twelve male LAC:P rats ~150 g body weight were randomly allocated to 6 groups. Group 1 was injected intrapleurally with 0.4 ml saline vehicle while under light ether anaesthesia. Groups 2 to 6 were each injected with 20 mg/0.4 ml of erionite suspension whilst similarly anaesthetised. After injection the animals were maintained in the normal way.

A further 2 animals, of similar weight, were injected intrapleurally with the short fibre version of the erionite.

Groups 1 and 2 were killed at 1 h after injection. Groups 3, 4, 5 and 6 were killed at 24, 48, 72 h and 7 days respectively. The animals inoculated with the short erionite were killed at 1 and 7 days.

Necropsies were performed on all animals. The lungs were distended with 10% buffered formalin and immersed in formalin for 48 h. After fixation, tissues were selected and processed for histological sectioning and paraffin wax sections cut at 5 $\mu$m.

Sections were also prepared from tissue obtained from a previous study where similar animals had been intrapleurally inoculated with the same erionite sample and had been allowed to survive longer, including seven animals that had developed mesothelioma. This tissue was sectioned and stained alongside the tissue from the short-term study.

9-Aminoacridine and propidium iodide were purchased from Sigma Chemical Company, St. Louis, MO, USA. A fresh normal rat liver was homogenised in isotonic saline and centrifuged on a bench centrifuge to produce a liver extract containing ~1 mg protein ml$^{-1}$. Sections of rat lung were dewaxed in the conventional manner prior to treatment with the aqueous extract of liver tissue for 18 h. Excess liver extract was carefully washed off the section and the treated sections then exposed to $10^{-3}$ M 9-aminoacridine for 2 min followed by 3 washes of 2 min each in three tanks of isotonic saline (Steven & Al-Ahmad, 1985). The sections were finally placed in $10^{-6}$ M propidium iodide for 1 min, rinsed and examined by fluorescent microscopy (Steven *et al.*, 1986*a*).

This fluorescent staining procedure results in cells with GB binding first 9-aminoacridine and then propidium iodide; as a consequence the cytoplasm and cell surface appear pink on a blue background. The nuclei of all cells bind propidium iodide to double stranded DNA, so in this study the nuclei should be ignored. We present data on the extranuclear staining of mesothelial cells lining the surface of the lung tissue of a series of rats exposed to erionite fibres. Those mesothelial cells with blue extranuclear staining lack GB whilst those mesothelial cells with pink extranuclear staining possess uninhibited GB (Steven *et al.*, 1986*a*).

### Results and discussion

When we employed 9-aminoacridine without propidium iodide, the mesothelial cells lining the surface of the lung tissue exhibited clearly visible yellow fluorescence in the extranuclear region. Under these conditions the nuclei remained unstained. We were unable to record these observations with satisfactory colour contrast on film due to the blue-green background. We therefore chose to increase the contrast of the extranuclear staining by co-stacking with propidium iodide which also caused pink fluorescent staining of the nuclei. Cells exhibiting pink staining throughout (e.g. the mesothelial cells) must therefore bind propidium iodide in both the nuclear and extranuclear regions. This result would be expected from the observations with 9-aminoacridine staining of mesothelial cells as described above.

We use the word extranuclear since it is not possible, in these sections, to define whether the 9-aminoacridine bound to the cytoplasmic or membrane bound guanidinobenzoatase. Other experiments with whole cultured cells have shown that at least part of the enzymic activity is associated with the external surface of these cells and is accessible to high molecular weight inhibitors of guanidinobenzoatase (FSS, unpublished data). In the present study, pretreatment of the sections with BZAR [an inhibitor of guanidinobenzoatase, Steven *et al.* (1988*b*)] prevented the binding of 9-aminoacridine to the extranuclear region of the mesothelial cells. Since BZAR is a rather specific inhibitor of guanidinobenzoatase we feel confident that the binding of 9-aminoacridine to these mesothelial cells of the treated rat lungs reflects the presence of this enzyme in these cells.

The sections of mesothelioma showed that virtually every tumour cell exhibited strong pink to red extranuclear fluorescence (Figure 1). The cells at the free edge of the tumour mass often seemed to be more intensely stained than those at the centre. The extranuclear staining indicated the presence of GB. All cell nuclei appeared pink due to the binding of propidium iodide to DNA. Bands of blue coloured connective tissues were seen, particularly in the sarcomatous type of tumour. The samples examined showed the full range of histological patterns associated with mesotheliomas, namely sarcomatous, epithelial and mixed cell types together with cystic, sclerotic and bone forming areas. There was no detectable difference in either the pattern or intensity of the red fluorescence in the various tumour types.

In contrast, sections from the group of animals injected with saline only showed no cytoplasmic or cell surface pink staining (Figure 2). The histological pattern was normal.

Sections from groups 2 to 6 showed a gradation of response. At 1 h after inoculation of erionite the mesothelial cells were large and 'humped' in appearance (Figure 3). They were blue in colour indicating lack of GB and there was an accumulation of cells in the submesothelial region. By 24 h this submesothelial accumulation of cells had increased. The mesothelial cells were still large and humped but some now exhibited pink extranuclear staining. At 48 h most of the mesothelial cells showed pink extranuclear staining (Figure 4). The mesothelial cells increased in number by 72 h and in some places were several cells thick. Nearly all these cells showed pink extranuclear staining. Below the pleural elastic lamina there was a marked accumulation of cells (Figure 5).

When the lesion had progressed 7 days, the pleura showed considerable thickening (Figure 6). The mesothelial cell layer was strongly positive for GB and was often several cells thick. Between the mesothelial cells and the elastic lamina was a band of connective tissue containing many spindle shaped cells possessing GB. Beneath the elastic lamina there were several foci of mononuclear cells, possible macrophages. Sections of lungs from animals examined between 7 and 140 days showed a similar pattern of pleural reaction and GB expression.

The animals injected with the short erionite showed little GB expression. Twenty-four hours after inoculation there were some inflammatory cells adhering to the mesothelium from the pleural cavity, but no evidence of change in the mesothelium or pleural layers. At 7 days it was possible to see an occasional mesothelial cell with pink staining cytoplasm but most were normal and there was no pleural thickening, hyperplasia or cell infiltrates.

We also examined the effect of an extract of rat lung tissue on these sections prior to fluorescent staining. The results were similar to those described for sections pretreated with the liver extract. The lung extract inhibited the binding of 9-aminoacridine to most cells within the lung tissue but significantly did not inhibit the binding of 9-aminoacridine to the mesothelial cells on the surface of the lung tissue.

The development of a fluorescent probe (9AA) for an enzyme associated with cells capable of migration provided a useful technique for the study of the pleura's early reaction to injury and enabled us to follow changes in this protease in mesothelial cells in the period following exposure to fibre.

If we consider the two extremes of the findings, firstly, the control animals showed no pink cytoplasm staining and so one can say that the cells in normal pleura do not express GB. At the other extreme the mesothelioma showed intense pink-red cytoplasmic staining indicating strong expression of GB. Mesotheliomas occur in several forms, from sarcomatous to epithelial cell types with a spectrum of mixed types in between. Each of these tumour types, when examined by this technique, exhibited strong GB expression as typified in Figure 1.

This study showed that changes occurred very rapidly in mesothelium following exposure to fibrous erionite. One hour after exposure morphological changes had occurred, the cells appeared 'humped' rather than flattened as in the control, although there was no expression of GB. After 24 h, however, the cytoplasm appeared pink indicating the presence of GB which enabled the cells to bind 9AA and PI outside the nucleus. With increase in time the number of mesothelial cells possessing GB increased, all cells in the much thickened mesothelial cell layer staining pink. The intensity and extent of this staining did not decline during the period of study. The many spindle cells in this thickened pleura may be reactive fibroblasts or more likely may be the multi-potential subserosal cell described by some workers (Bolen *et al.*, 1987). These authors describe these cells as having the ultrastructural features of myofibroblasts which can change their morphology and intermediate filament expression as they approximate the serosal surface. Our study indicates that these cells respond to the stimulus of the fibre in the pleural cavity by expressing GB and that they retain this expression.

In contrast, the short erionite produced very little GB expression. In spite of being the same chemical injected at the same site there was clearly a different sequence of events. There was no pleural thickening, mesothelial hyperplasia or cell infiltrate. Only very occasional pink stained cells could be found in the mesothelium after 7 days. This model indicates that there is a similar correlation between size/shape of mineral particles and GB expression as there is with mesothelioma formation (Stanton *et al.*, 1977; J.C. Wagner, personal communication).

Figure 1

Figure 2

Figure 3

Figure 4

Figure 5

Figure 6

**Figure 1** Section of mesothelioma after fibrous erionite inoculation. The tumour cells exhibit intense pink staining. (9AA, PI, ×250).

**Figure 2** Section of control rat lung showing visceral pleura. The cell nuclei appear pink but the cytoplasm of all cells is blue. (9AA, PI, ×250).

**Figure 3** Visceral pleura, one hour after intrapleural inoculation of fibrous erionite. The mesothelial cells are enlarged but still show blue cytoplasm. (9AA, PI, ×250).

**Figure 4** Visceral pleura, forty-eight hours after intrapleural inoculation of fibrous erionite. Most mesothelial cells show pink cytoplasmic staining. (9AA, PI, ×250).

**Figure 5** Visceral pleural, seventy-two hours after intrapleural inoculation of fibrous erionite. Mesothelial hyperplasia with all cells showing pink cytoplasmic staining. Beneath the blue staining elastic lamina there is a marked accumulation of cells with pink cytoplasm. (9AA, PI, ×250).

**Figure 6** Visceral pleura, seven days after inoculation of fibrous erionite. The mesothelial cells show intense pink staining. There is marked thickening of the pleura, with many pink staining spindle shaped cells. (9AA, PI, ×250).

We have concentrated here upon the cells of the pleura, in particular the mesothelial cells, as it is from the pleura that mesotheliomas develop. It is apparent that there are aggregates of GB positive cells in the alveoli immediately beneath the pleural elastic lamina, these cells await further study.

There is no reason to suppose that the expression of GB is specific to erionite treatment, indeed preliminary studies by us indicate that GB expression can be seen following implantation of asbestos (unpublished data).

Preliminary trials by us also indicate that formalin fixed paraffin processed human mesothelioma tissue exhibits strong GB expression similar to that seen in Figure 1 (unpublished data). It is of interest that the fluorogenic substrate for trypsin-like enzymes, recently introduced by Leytus *et al.* (1983) is now known to be an effective inhibitor of GB (Steven *et al.*, 1988*b*). Such inhibitors might possibly have a role in the suppression of tumours such as mesothelioma.

FSS wishes to thank both the Cancer Research Campaign and the Imperial Cancer Research Fund for generous financial support during the duration of these studies. We thank Mrs R. Hill for typing the manuscript.

### References

BARIS, Y.I., SAHIN, A.A., OZESMI, M. & 5 others (1978). An outbreak of pleural mesothelioma and chronic fibrosing pleurisy in the village of Karain/Urgup in Anatolia. *Thorax*, **33**, 181.

BOLEN, J.W., HAMMAR, S.P. & McNUTT, M.A. (1987). Serosal tissue: Reactive tissue as a model for understanding mesotheliomas. *Ultrastruct. Path.*, **11**, 251.

HILL, R.J., EDWARDS, R.E. & DRIVER, H.E. (1987). Early changes following the intrapleural inoculation of the mineral fibre erionite. *Hum. Toxicol.*, **6**, 435.

LEYTUS, S., MELHADO, L.L. & MANGEL, W.F. (1983). Rhodamine-based compounds as fluorogenic substrates for serine proteases. *Biochem. J.*, **209**, 299.

POOLEY, F.D. (1979). Evaluation of fibre samples taken from the vicinity of two villages in Turkey. In *Dust and Disease*, Lemen & Dement (eds) p. 41. Pathotox Publishers: Park Forest South Illinois.

STANTON, M.D., LAYARD, M., TEGERIS, A., MILLER, E., MAY, M. & KENT, E. (1977). Carcinogenicity of fibrous glass, pleural response in the rat relationship to fibre dimension. *J. Natl Cancer Inst.*, **58**, 587.

STEVEN, F.S. & AL-AHMAD, R.K. (1983). Evidence for an enzyme which cleaves the guanidinobenzoate moiety from active-site titrants specifically designed to inhibit and quantify trypsin. *Eur. J. Biochem.*, **130**, 335.

STEVEN, F.S., GRIFFIN, M.M. & AL-AHMAD, R.K. (1985). The design of fluorescent probes which bind to the active centre of guanidinobenzoatase. *Eur. J. Biochem.*, **149**, 35.

STEVEN, F.S., GRIFFIN, M.M. & AL-AHMAD, R.K. (1986*a*). Design fluorescent probes for an enzyme on the surface of tumour cells. *J. Chromatography* (*Biomedical Applications*), **376**, 211.

STEVEN, F.S., GRIFFIN, M.M., WONG, T.L.H. & ITZHAKI, S. (1986*b*). Evidence for inhibitors of the cell surface protease guanidinobenzoatase. *J. Enz. Inhib.*, **1**, 127.

STEVEN, F.S., GRIFFIN, M.M., FREEMONT, A.J. & JOHNSON, J. (1988*a*). Inhibition of guanidinobenzoatase: Evidence for multiple forms of this protease on different tumour cells. *J. Enz. Inhib.* (in press).

STEVEN, F.S., GRIFFIN, M.M., MANGEL, W.F., MAIER, H. & ALTMANNSBERGER, M. (1988*b*). Inhibition of guanidinobenzoatase by a substrate for trypsin-like enzymes. *J. Enz. Inhib.*, **2**, 209.

WAGNER, J.C., SKIDMORE, J.W., HILL, R.J. & GRIFFITHS, D.M. (1985). Erionite exposure and mesotheliomas in rats. *Br. J. Cancer*, **51**, 727.